# Increased *O*-GlcNAcylation of Endothelial Nitric Oxide Synthase Compromises the Anti-contractile Properties of Perivascular Adipose Tissue in Metabolic Syndrome

**DOI:** 10.3389/fphys.2018.00341

**Published:** 2018-04-06

**Authors:** Rafael M. da Costa, Josiane F. da Silva, Juliano V. Alves, Thiago B. Dias, Diane M. Rassi, Luis V. Garcia, Núbia de Souza Lobato, Rita C. Tostes

**Affiliations:** ^1^Department of Pharmacology, Ribeirao Preto Medical School, University of Sao Paulo, Ribeirao Preto, Brazil; ^2^Department of Biomechanics, Medicine and Locomotive Apparatus Rehabilitation, Ribeirao Preto Medical School, University of Sao Paulo, Ribeirao Preto, Brazil; ^3^Department of Physiology, Institute of Health Sciences, Federal University of Goias, Jatai, Brazil

**Keywords:** PVAT, *O*-GlcNAc, high-sugar diet, eNOS, vascular function

## Abstract

Under physiological conditions, the perivascular adipose tissue (PVAT) negatively modulates vascular contractility. This property is lost in experimental and human obesity and in the metabolic syndrome, indicating that changes in PVAT function may contribute to vascular dysfunction associated with increased body weight and hyperglycemia. The *O*-linked β-N-acetylglucosamine (*O*-GlcNAc) modification of proteins (*O*-GlcNAcylation) is a unique posttranslational process that integrates glucose metabolism with intracellular protein activity. Increased flux of glucose through the hexosamine biosynthetic pathway and the consequent increase in tissue-specific *O*-GlcNAc modification of proteins have been linked to multiple facets of vascular dysfunction in diabetes and other pathological conditions. We hypothesized that chronic consumption of glucose, a condition that progresses to metabolic syndrome, leads to increased *O*-GlcNAc modification of proteins in the PVAT, decreasing its anti-contractile effects. Therefore, the current study was devised to determine whether a high-sugar diet increases *O*-GlcNAcylation in the PVAT and how increased *O*-GlcNAc interferes with PVAT vasorelaxant function. To assess molecular mechanisms by which *O*-GlcNAc contributes to PVAT dysfunction, thoracic aortas surrounded by PVAT were isolated from Wistar rats fed either a control or high sugar diet, for 10 and 12 weeks. Rats chronically fed a high sugar diet exhibited metabolic syndrome features, increased *O*-GlcNAcylated-proteins in the PVAT and loss of PVAT anti-contractile effect. PVAT from high sugar diet-fed rats for 12 weeks exhibited decreased NO formation, reduced expression of endothelial nitric oxide synthase (eNOS) and increased *O*-GlcNAcylation of eNOS. High sugar diet also decreased OGA activity and increased superoxide anion generation in the PVAT. Visceral adipose tissue samples from hyperglycemic patients showed increased levels of *O*-GlcNAc-modified proteins, increased ROS generation and decreased OGA activity. These data indicate that *O*-GlcNAcylation contributes to metabolic syndrome-induced PVAT dysfunction and that *O*-GlcNAcylation of eNOS may be targeted in the development of novel therapies for vascular dysfunction in conditions associated with hyperglycemia.

## Introduction

The perivascular adipose tissue (PVAT) is a highly active endocrine organ that releases a wide variety of adipokines that control vascular smooth muscle tone in veins, conductance arteries, and vessels of smaller caliber (Szasz and Webb, 2012). The vasoactive factors released by the PVAT (Adventitium-Derived Relaxing Factors—ADRF) under physiological conditions include adiponectin, angiotensin- (1-7), hydrogen peroxide, leptin, nitric oxide (NO), and other agents that negatively modulate vascular contraction, that is, the PVAT exerts an anti-contractile effect and is essential for the maintenance of vascular function (Fernandez-Alfonso et al., 2013; Xia and Li, 2017).

In obesity and metabolic syndrome, there is an increase in the amount of PVAT accompanied by changes in the PVAT pattern of adipokines expression, infiltration, and activation of circulating inflammatory cells (Lumeng et al., 2007), hypoxia, and oxidative stress (Gao, 2007). The net effect of these changes is a profound impairment of the vasoactive properties of PVAT, leading to an imbalance in favor of PVAT-derived vasoconstrictor substances, as well as changes in their signaling pathways in the vasculature (da Costa et al., 2017). The predominant mechanisms leading to a dysfunctional PVAT in obesity and metabolic syndrome have not been identified so far.

Post-translational modifications of intrinsic components of PVAT seem to contribute to the loss of its anti-contractile effect in metabolic and cardiovascular diseases. As an example, reduced NO synthase phosphorylation in the serine activation residue (Ser^1177^) and decreased phosphorylation of adenosine monophosphate-activated protein kinases, which positively regulate NO synthase activity, have been reported in the PVAT of animals fed a high fat diet for 6 months (Ma et al., 2010).

Among the many types of post-translational modifications, a great deal of interest has been directed to *O*-GlcNAcylation, i.e., glycosylation with *N*-acetyl-glucosamine (*O*-GlcNAc), which occurs by the addition of *N*-acetyl-glucosamine to the oxygen of the hydroxyl group of serine and threonine residues. Various cytoplasmic and nuclear proteins, including kinases, phosphatases, transcription factors, and cytoskeletal proteins are targets of *O*-GlcNAcylation (Hart et al., 2007). The activity of the enzymes *O*-GlcNAc transferase (OGT) and *O*-GlcNAcase (OGA) directly control the *O*-GlcNAc process. Whereas, OGT catalyzes the addition of *N*-acetyl-glucosamine in the target proteins, the enzyme OGA catalyzes the hydrolytic removal of *O*-GlcNAc (Lima et al., 2009). It is noteworthy that both enzymes are themselves targeted for *O*-GlcNAcylation and other post-translational modifications (Laczy et al., 2009).

*O*-GlcNAcylation integrates glucose metabolism with the activity of innumerable intracellular proteins. Increased glucose flow through the hexosamine biosynthesis pathway and increased *O*-GlcNAc modification of proteins are associated with the multiple facets of vascular dysfunction in diabetes mellitus and obesity, including endothelial function impairment, inflammation, fibrosis, and metabolic dysfunction (Lima et al., 2009). However, the contribution of *O*-GlcNAcylation to PVAT dysfunction in metabolic syndrome has not been determined. Thus, we hypothesized that increased *O*-GlcNAcylation of endothelial NO synthase (eNOS) in the PVAT produces loss of its anti-contractile effect leading to PVAT and vascular dysfunctions in metabolic syndrome. In the present study, we sought to determine whether chronic high sugar diet and the subsequent metabolic syndrome increase *O*-GlcNAcylation in the PVAT and how increased *O*-GlcNAc interferes with PVAT vasorelaxant function.

## Materials and methods

### *In vivo* studies

All experimental protocols were performed in accordance with the National Council for Animal Experimentation Control (CONCEA) and were approved by the Ethics Committee on Animal Use (CEUA) of the University of Sao Paulo, Ribeirao Preto, Brazil (Protocol n° 206/2016).

Six week-old male Wistar rats were obtained from the University of Sao Paulo, Ribeirao Preto, Brazil and maintained in the Animal Facility of the Pharmacology Department, Ribeirao Preto Medical School, on 12-h light/dark cycles under controlled temperature (22 ± 1°C) with *ad libitum* access to food and water. After a 1-week acclimatization period, rats were randomly divided into two groups: (1) rats maintained on a control diet; (2) rats receiving a high sugar diet for 10 or 12 weeks. The high sugar diet consisted of 33% control diet (Nuvilab® CR1, Nuvital, Brazil), 33% condensed milk, and 7% sucrose by weight, the remaining being water (Silva et al., 2016). The energy density was 12.26 kJ/g for the control diet and 13.35 kJ/g for the high sugar diet. After the treatment period, rats were killed by carbon dioxide (CO_2_) inhalation.

### Humans samples

This study was approved by the Research Ethics Committee of the University of Sao Paulo (Protocol n° 14189/2012) and all the stated rules for human research were followed. Visceral adipose tissue was taken from portions of the omentum from patients with bowel cancer submitted to surgical procedures at the Clinics Hospital of the Ribeirao Preto Medical School at the University of Sao Paulo (8 male patients, 42–75 years-old). All patients provided signed informed and written consent prior to participation in this study. The specimens were taken from macroscopically normal tissue, and care was taken to exclude adipose tissue supplying the area of the tumor. Patients were divided into the following groups: normoglycemic patients (blood glucose lower than 100 mg/dL) and chronic hyperglycemic patients (blood glucose over 150 mg/dL). Visceral adipose tissue is directly associated with the function of resistance arteries (Farb et al., 2012) and allows the investigation of the involvement of these deposits on vascular function. The use of human adipose tissue surrounding the aorta, which would better correlate with the animal studies, was not possible.

### Biochemical profile of rats fed the control and high sugar diets

Glucose levels were determined using a glucose analyzer (Accu-Check, Roche Diagnostics, Brazil). In addition, total cholesterol and triglycerides concentrations were determined in serum samples from rats fasted for 12 h, by an enzymatic colorimetric method (Doles®, Brazil). Serum insulin concentration (ng/mL) was determined by radioimmunoassay (Insulin Kit®, Brazil). Insulin sensitivity was calculated using the HOMA-IR index (Homeostasis Model Assessment) (Pan et al., 2015), which takes into account insulin and fasting blood glucose levels, using the following mathematical formula: HOMA-IR = fasting insulin x fasting glucose/22.5. The oral glucose tolerance test (OGTT) was performed to evaluate glucose tolerance. Rats were deprived of food for 6 h. Blood was sampled from the caudal vein immediately before (baseline, *t*_0_) and after (*t*_15_*, t*_30_*, t*_60_*, t*_90_*, t*_120_ minutes) administration of 2 g of glucose/kg by oral gavage.

### Assessment of vascular function

Thoracic aorta was rapidly removed, transferred to an ice-cold (4°C) Krebs Henseleit modified solution [(in mM) 130 NaCl, 14.9 NaHCO_3_, 4.7 KCl, 1.18 KH_2_PO_4_, 1.17 MgSO_4_· 7H_2_O, 5.5 glucose, 1.56 CaCl_2_·2H_2_O and 0.026 EDTA] gassed with 5% CO_2_ / 95% O_2_ to maintain a pH of 7.4, and dissected into 3 mm rings whereby perivascular fat and connective tissues were either removed (PVAT–) or left intact (PVAT+). Aortic rings were mounted in a wire myograph to measure isometric tension, as previously described (Costa et al., 2016). Vessels were allowed to equilibrate for about 30 min in Krebs Henseleit solution and baseline tension of 30 mN. After the stabilization period, the arteries were stimulated with Krebs solution containing a high concentration of potassium [K^+^ (120 mM)] to evaluate the contractile capacity of the segments. KCl-mediated contraction responses were similar in all experimental conditions (~35 mN). Endothelial function was assessed by testing the relaxant effect of acetylcholine (ACh, 10^−6^ M) on vessels contracted with phenylephrine (PE, 10^−7^ M). In experiments with endothelium-denuded vessels, aortic rings were subjected to rubbing of the intimal surface. Rings showing a maximum of 5% relaxation in response to ACh were considered to be without endothelium. Cumulative concentration-response curves to PE (10^−10^−10^−4^ M) and sodium nitroprusside (SNP, 10^−10^−10^−4^ M) were performed in PVAT (+) or PVAT (–) aortic rings. Contractile responses to PE were also determined after incubation with L-NAME (10^−4^ M), nitric oxide synthase inhibitor, 30 min before adding the contractile agonist. Each vascular preparation was tested with a single agent.

### PVAT *ex vivo* incubation

PVAT from rats fed the control diet were incubated for 24 h in normoglycemic (5.6 mM glucose) Dulbecco's Modified Eagle Medium (DMEM), in the presence or absence of the OGA inhibitor Thiamet G (10^−6^ M, Okuda, 2017). PVAT from rats fed the control diet were incubated for 3, 6, and 12 h in hyperglycemic (25 mM glucose) DMEM. In this experiment, the control was obtained using PVAT incubated in normoglycemic DMEM for 12 h. In addition, PVAT from rats fed the control diet were incubated for 6 h in hyperglycemic DMEM in the presence or absence of the superoxide anion (${\text{O}}_{2}^{.-}$) scavenger Tiron (10^−4^ M, Alves-Lopes et al., 2016). For this condition, the experimental control was PVAT incubated in normoglycemic DMEM for 6 h. During incubations, PVAT was individually maintained in six-well Petri dishes at 37°C and 5% CO_2_.

### Western blot analysis

PVAT from thoracic aorta and visceral adipose tissue from humans were frozen in liquid nitrogen and homogenized in a lysis buffer [50 mM Tris/HCl, 150 mM NaCl, 1% Nonidet P40, 1 mM EDTA, 1 μg/ml leupeptin, 1 μg/ml pepstatin, 1 μg/ml aprotinin, 1 mM sodium orthovanadate, 1 mM phenylmethanesulfonyl fluoride (PMSF), and 1 mM sodium fluoride]. The tissue extracts were centrifuged, and total protein content was quantified using the Bradford method (Bradford, 1976). Proteins (20 μg) were separated by electrophoresis on 10% polyacrylamide gel, and transferred on to nitrocellulose membranes. Non-specific binding sites were blocked with 5% bovine serum albumin (BSA) in Tris buffered saline (TBS) containing 0.1% Tween 20 (for 1 h at 24°C). Membranes were incubated with antibodies (at the indicated dilutions) overnight at 4°C. Antibodies were used as follows: anti-Anti-β-*O*-Linked *N*-Acetylglucosamine (1:5000 dilution; Sigma-Aldrich Inc., Germany), anti-OGA (1:1000 dilution; Sigma-Aldrich Inc., Germany), anti-OGT (1:1000 dilution; Abcam, UK), anti-GAPDH (1:10000 dilution; Sigma-Aldrich Inc., Germany). After incubation with secondary antibodies, signals were obtained by chemiluminescence and quantified densitometrically.

### OGA activity in rat PVAT and visceral adipose tissue of humans

The tissue proteins were extracted according to the previous item and quantified using the Bradford method. Proteins (3 μg) were eluted in 100 μL of citrate buffer (0.05 M, pH 5.0). After elution, the OGA substrate 4-Methylumbelliferyl *N*-acetyl-β-D-glucosaminide (4-MUNAG, 300 μg/ml, Sigma-Aldrich Inc., Germany) and OGA inhibitor Thiamet G (10^−6^ M) were added. The reaction occurred in an oven at 37°C for 30 min. Then, the reaction was stopped after addition glycine buffer (0.1 M, pH 12). The breaking of the 4-MUNAG by the OGA emits fluorescence after excitation of 362 nm and emission of 448 nm. The values were expressed in fluorescence relative units through the delta of the reactions in the presence and absence of Thiamet G.

### Immunoprecipitation

To evaluate the degree *O*-GlcNAc modification on eNOS, direct immunoprecipitation was performed using magnetic beads, according to the manufacturer protocol (PureProteome Protein G Magnetic Beads, LSKMAGG02, Merck-Millipore, UK). Briefly, the bead-antibody complex was formed by incubating 30 μL of beads with 5 μg of anti-eNOS antibody (Sc-376751-Santa Cruz Biotechnology, USA). Subsequently, the bead-antibody complex was added to 500 μg of proteins derived from the total protein extract of the PVAT of the animals. After overnight incubation at 4°C, the bead-antibody-protein complex was precipitated with the aid of the magnetic tube rack (DynaMag TM-2, Invitrogen^TM^, Thermo Fisher Scientific Inc., UK). Then, proteins were separated by sodium dodecyl sulfate/polyacrylamide gel (8%) electrophoresis (SDS/PAGE), as described in the western blot protocol. Anti-*O*-GlcNAc (O7764, Sigma-Aldrich Inc., Germany) and anti-eNOS (Sc-376751, Santa Cruz Biotechnology, USA) primary antibodies were used. Protein bands were detected by chemiluminescence reaction (Luminata Forte, WBLUF0100, Merck-Millipore, UK) and the intensity of the bands was evaluated by densitometric analysis using ImageQuant software. The results are expressed by the ratio of the intensity of the *O*-GlcNAc bands to the intensity of the respective bands of the immunoprecipitated eNOS.

### Measurement of reactive oxygen species

#### Dihydroethidium

ROS generation in PVAT was assessed by dihydroethidium (DHE), as previously described (Suzuki et al., 1995). Aortas surrounded by periaortic fat were embedded in medium for frozen tissue specimens to ensure optimal cutting temperature (OCT™) and stored at −80°C. Fresh-frozen specimens were cross-sectioned at 10 μm thickness and placed on slides covered with poly-(L-lysine) solution. The tissue was loaded with the non-selective dye for ROS detection DHE (5 × 10^−6^ M; for 30 min at 37°C), which was prepared in phosphate buffer 0.1 M. Images were collected on a ZEISS microscope and the results are expressed as fold changes relatively to the control. Fluorescent images were analyzed by measuring the mean optical density of the fluorescence in a computer system (Image J software^©^) and normalized by the area.

#### Lucigenin

ROS generation in the PVAT and humans visceral adipose tissue was measured by a luminescence assay using lucigenin as the electron acceptor and NADH as the substrate. Periaortic fat from control and high sugar diet-fed rats was homogenized in assay buffer (50 mM KH_2_PO_4_, 1 mM EGTA, and 150 mM sucrose, pH 7.4) with a glass-to-glass homogenizer. The assay was performed with 100 μL of sample, lucigenin (5 μM), NADH (0.1 mM), and assay buffer. Luminescence was measured for 30 cycles of 18 s each by a luminometer (Lumistar Galaxy, BMG Labtechnologies, Ortenberg, Germany). Basal readings were obtained prior to the addition of NADH and the reaction was started by the addition of the substrate. Basal and buffer blank values were subtracted from the NADH-derived luminescence. ${\text{O}}_{2}^{.-}$ was expressed as relative luminescence units (RLU)/mg of protein.

### Nitric oxide metabolites levels

PVAT from thoracic aorta and human visceral adipose tissue were immediately frozen in liquid nitrogen, pulverized and homogenized in 20 mM Tris-HCl (pH 7.4). The samples were centrifuged (5,000 × g for 10 min at 4°C) and the total protein content was quantified using the Bradford method. The samples were analyzed in duplicate for nitrite and nitrate (NOx) using ozone-based chemiluminescence assay. Briefly, PVAT samples were treated with cold ethanol (1:2 mesenteric bed to ethanol, for 30 min at −20°C) and centrifuged (4,000 × g for 10 min). NOx levels were measured by injecting 25 μL of supernatant in a container vent glass containing 0.8% of vanadium (III) in HCl (1 N) at 90°C, which reduces NOx into NO gas. A stream of nitrogen was bubbled through the purge vessel containing vanadium (III) with sodium hydroxide [NaOH (1N)], and then through an analyzer (Sievers Nitric Oxide Analyzer® 280, GE Analytical Instruments, Boulder, CO, USA).

### Data and statistical analyses

Data are expressed as mean ± SEM. Concentration-response curves were fitted using the function Y = Bottom + [Top-Bottom]/[1+10^∧^(LogEC50-X)] into a curve by non-linear regression analysis. The curves were compared using the delta of the areas under curves, maximal response (Emax) and *p*D_2_ (defined as the negative logarithm of the EC_50_ values). The delta of the areas under curves was calculated as the difference between the concentration-response curves in the presence or in the absence of the PVAT and different drugs. Student's *t*-test was used to compare the delta of the areas under curves. Two-way ANOVA with Bonferroni post-test was used to compare Emax, *p*D_2_, biochemical, and high glucose experiments. Mann Whitney test was used to compare groups with *n* = 4. The Prism software, version 5.0 (GraphPad Software Inc., San. Diego, CA, USA) was used to analyze these parameters. N represents the number of animals used. *p*-values < 0.05 were considered significant.

## Results

**Figure 1:**
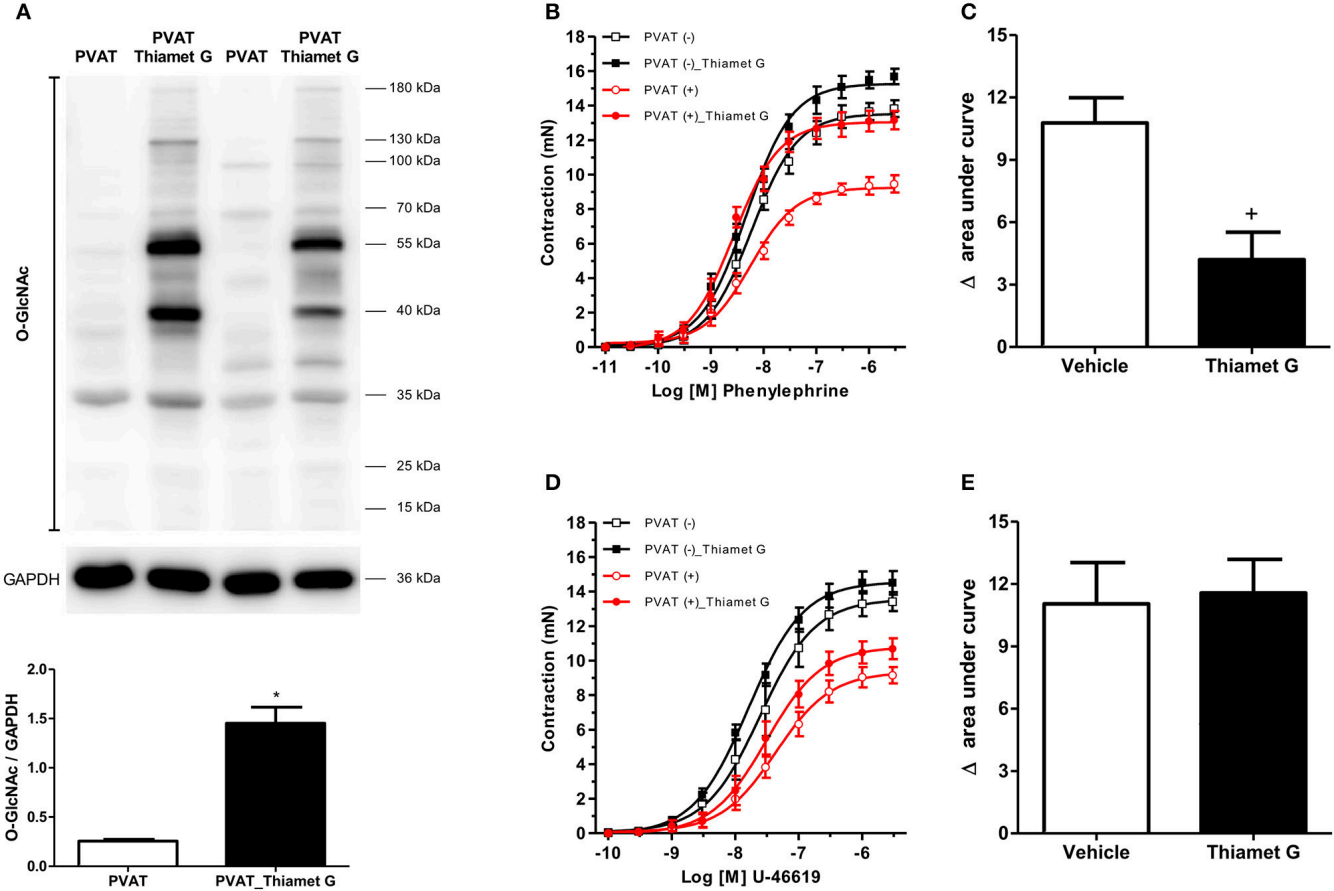
Increased *O*-GlcNAc-modified proteins induce loss of the PVAT anti-contractile effect**. (A)** Expression of *O*-GlcNAc-modified proteins in PVAT incubated with the *O*-GlcNAcase (OGA) inhibitor, Thiamet G (10^−6^ M, for 24 h, *n* = 6) or vehicle (*n* = 6). Concentration-response curves to **(B)** phenylephrine (*n* = 7) and **(D)** thromboxane A_2_ receptor agonist, U-46619 (*n* = 7), were performed in endothelium-denuded aortic rings without (–) or with (+) PVAT, incubated with Thiamet G (10^−6^ M, for 24 h) or vehicle. **(C,E)** show the difference (delta) of the areas under the curves to phenylephrine and U-46619, respectively, in the absence and presence of PVAT after incubation with Thiamet G or vehicle. Data are represented as the mean ± SEM. Student's *t*-test: ^*^p < 0.05 PVAT_Thiamet *G* vs. PVAT; ^+^*p* < 0.05 Thiamet G vs. Vehicle.

To determine the effects of increased *O*-GlcNAc-modified proteins on PVAT function, *O*-GlcNAcylation of rat thoracic aorta PVAT proteins was induced using Thiamet G (10^−6^ M for 24 h), a potent and selective *O*-GlcNAcase (OGA) inhibitor. Thiamet G significantly increased the expression of *O*-GlcNAc-modified proteins in the PVAT (Figure 1A). To determine whether increased levels of *O*-GlcNAc-proteins reduce the PVAT anti-contractile effect, endothelium-denuded thoracic aortic rings with or without PVAT were incubated with Thiamet G (10^−6^ M for 24 h) or vehicle. Subsequently, concentration-response curves to either phenylephrine or the thromboxane A_2_ receptor agonist U-46619 were performed. Emax values showed, as expected, that the presence of PVAT reduced the contraction to phenylephrine and U-46619 in aortic rings incubated with vehicle (Table 1). Analysis of the delta of the areas under curves showed that incubation of PVAT-intact aortic rings with Thiamet G decreased the PVAT anti-contractile effect in arteries contracted with phenylephrine (Figures 1B,C), but not in arteries contracted with U-46619 (Figures 1D,E).

**Table 1:** *p*D_2_ and Emax (mN) values of phenylephrine and U-46619-induced contraction in thoracic aorta arteries incubated with vehicle or Thiamet G.

	***p*****D**_**2**_		**Emax**	
**Groups**	**Phenylephrine**	**U-46619**	**Phenylephrine**	**U-46619**
PVAT (–)	8.2 ± 0.05 (*n* = 6)	7.6 ± 0.09 (*n* = 7)	13.5 ± 0.2 (*n* = 6)	13.5 ± 0.5 (*n* = 7)
PVAT (–)_Thiamet G	8.3 ± 0.05 (*n* = 6)	7.7 ± 0.05 (*n* = 7)	15.2 ± 0.2 (*n* = 6)	14.5 ± 0.2 (*n* = 7)
PVAT (+)	8.2 ± 0.07 (*n* = 6)	7.4 ± 0.08 (*n* = 7)	9.2 ± 0.2 (*n* = 6)^*^	9.3 ± 0.3 (*n* = 7)^*^
PVAT (+)_Thiamet G	8.6 ± 0.06 (*n* = 6)^+^	7.5 ± 0.08 (*n* = 7)	13.0 ± 0.2 (*n* = 6)^+^	10.8 ± 0.4 (*n* = 7)^+^

Data represent the mean ± SEM of n experiments. Two-way ANOVA:

* p < 0.05 vs. respective PVAT (–);

+ *p < 0.05 vs. respective PVAT (+)*.

**Figure 2:**
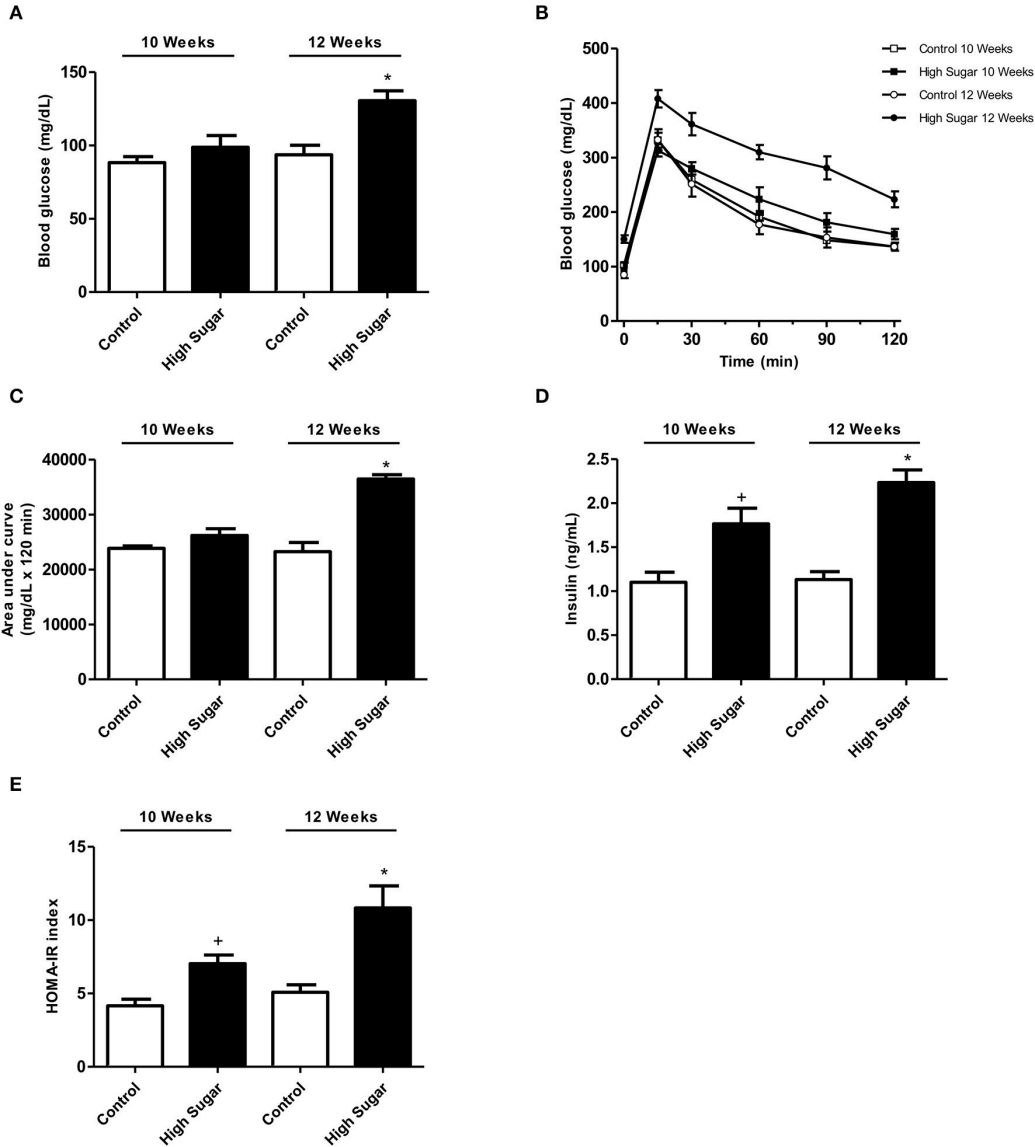
High sugar diet-induced disturbs in the rat glucose metabolism. **(A)** Glucose serum levels in rats fed the control (*n* = 8) or high sugar (*n* = 8) diet for 10 or 12 weeks. **(B)** Glucose serum levels and **(C)** the areas under the curves obtained during the OGTT in rats fed the control (*n* = 8) or high sugar (*n* = 8) diet for 10 or 12 weeks. **(D)** Insulin serum levels in rats fed the control (*n* = 8) or high sugar (*n* = 8) diet for 10 or 12 weeks. **(E)** HOMA-IR index in rats fed with control (*n* = 8) or high sugar (*n* = 8) diet for 10 or 12 weeks. Data are represented as the mean ± SEM. Two-way ANOVA: ^+^*p* < 0.05 High Sugar 10 weeks vs. Control 10 weeks; ^*^p < 0.05 High Sugar 12 weeks vs. Control 12 weeks.

Considering that *O*-GlcNAcylation integrates glucose metabolism with intracellular protein activity and mediates multiple mechanisms of vascular dysfunction in metabolic syndrome, including impaired contractility (Lima et al., 2016), the possible relevance of PVAT *O*-GlcNAcylation following a high sugar diet was assessed. Rats fed the high sugar diet for 10 and 12 weeks exhibited an increase in all anthropometric parameters, total cholesterol and triglycerides (Table 2) compared with rats on the control diet. Only rats fed the high sugar diet for 12 weeks showed increased serum glucose levels (Figure 2A) and glucose intolerance, determined by the areas under the curves in the OGTT (Figures 2B,C). Insulin serum levels (Figure 2D) and HOMA-IR index (Figure 2E) were increased in rats fed the high sugar diet for 10 and 12 weeks.

**Table 2:** Characteristics of rats fed the Control and High Sugar diets.

	**Control 10 Weeks**	**High sugar 10 Weeks**	**Control 12 Weeks**	**High sugar 12 Weeks**
Initial body mass (g)	252.6 ± 2.0	254.1 ± 2.3	253.2 ± 2.2	255.3 ± 3.2
Final body mass (g)	469.4 ± 3.0	583.2 ± 3.7^+^	493.9 ± 3.1	657.5 ± 3.9^*^
Body mass gain (g)	216.8 ± 2.5	329.1 ± 3.1^+^	240.7 ± 2.6	402.2 ± 3.1^*^
Epididymal fat (g)	5.32 ± 0.1	9.57 ± 0.6^+^	5.89 ± 0.2	11.73 ± 0.6^*^
Visceral fat (g)	6.87 ± 0.3	12.36 ± 0.5^+^	7.33 ± 0.1	15.18 ± 0.6^*^
Retroperitoneal fat (g)	6.36 ± 0.2	11.62 ± 0.4^+^	7.19 ± 0.2	14.25 ± 0.5^*^
Adiposity index (%)	3.97 ± 0.1	5.75 ± 0.1^+^	4.13 ± 0.1	6.26 ± 0.1^*^
Total cholesterol (mg/dL)	88.7 ± 5.2	113.8 ± 2.1^+^	90.2 ± 4.8	124.2 ± 3.7^*^
Triglycerides (mg/dL)	68.2 ± 2.3	83.4 ± 3.1^+^	67.1 ± 3.9	92.8 ± 2.8^*^

Data represent the mean ± SEM of n experiments. Two-way ANOVA:

+ p < 0.05 vs. Control 10 Weeks;

* *p < 0.05 vs. Control 12 Weeks*.

**Figure 3:**
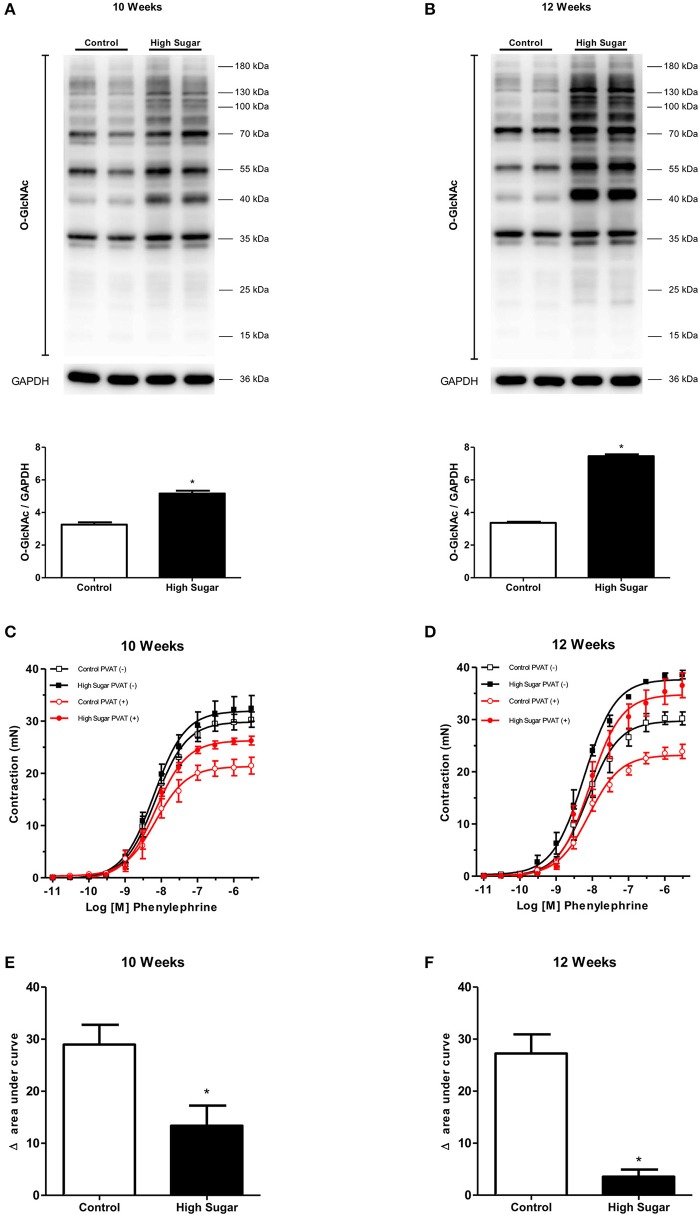
High sugar diet increases *O*-GlcNAc-modified proteins in the PVAT and impairs PVAT anti-contractile effect. Expression of *O*-GlcNAc-modified proteins in thoracic aorta PVAT of rats fed the control (*n* = 6) or high sugar (*n* = 6) diet for **(A)** 10 or **(B)** 12 weeks. Concentration-response curves to phenylephrine were performed in endothelium-denuded aortic rings, without (–) or with (+) PVAT, from rats fed the control (*n* = 7) or high sugar (*n* = 7) diet for **(C)** 10 or **(D)** 12 weeks. **(E**,**F)** show the delta of the areas under the curves to phenylephrine, in the absence and presence of PVAT, in arteries of rats fed the control or high sugar diet for 10 or 12 weeks, respectively. Data are represented as the mean ± SEM. Student's *t*-test: ^*^p < 0.05 High Sugar vs. Control.

To test the hypothesis that the high sugar diet increases PVAT *O*-GlcNAc levels leading to the loss of its anti-contractile effect, the content of *O*-GlcNAc-modified proteins in the PVAT from rats fed either the control or high sugar diet was determined. *O*-GlcNAcylated-proteins content was significantly increased in the PVAT from rats on the high sugar diet for 10 (Figure 3A) and 12 (Figure 3B) weeks. To determine the effects of high sugar diet on PVAT function, concentration-response curves to phenylephrine were performed in endothelium-denuded aortic rings with or without PVAT. Emax values showed that the presence of PVAT reduced the contraction to phenylephrine in arteries from rats fed the control diet, regardless of the duration of the diet (Table 3). Analysis of the delta of the areas under the curves confirmed that high sugar diet decreased the PVAT anti-contractile effect in arteries from rats fed for 10 (Figures 3C,E) or 12 (Figures 3D,F) weeks.

**Table 3:** *p*D_2_ and Emax (mN) values of phenylephrine-induced contraction in thoracic aorta arteries from Control or High Sugar diet-fed rats.

	***p*****D**_**2**_		**Emax**	
**Groups**	**10 Weeks**	**12 Weeks**	**10 Weeks**	**12 Weeks**
Control PVAT (–)	8.1 ± 0.06 (*n* = 6)	8.1 ± 0.07 (*n* = 6)	29.8 ± 0.6 (*n* = 6)	29.7 ± 0.8 (*n* = 6)
High Sugar PVAT (–)	8.2 ± 0.07 (*n* = 7)	8.1 ± 0.05 (*n* = 7)	31.9 ± 0.8 (*n* = 7)	37.6 ± 0.6 (*n* = 7)
Control PVAT (+)	8.1 ± 0.09 (*n* = 6)	8.0 ± 0.04 (*n* = 6)	21.3 ± 0.7 (*n* = 6)^*^	23.1 ± 0.4 (*n* = 6)^*^
High Sugar PVAT (+)	8.1 ± 0.04 (*n* = 7)	8.3 ± 0.07 (*n* = 7)^+^	26.2 ± 0.4 (*n* = 7)^+^	34.7 ± 0.8 (*n* = 7)^+^

Data represent the mean ± SEM of n experiments. Two-way ANOVA:

* p < 0.05 vs. respective Control PVAT (–);

+ *p < 0.05 vs. respective Control PVAT (+)*.

**Figure 4:**
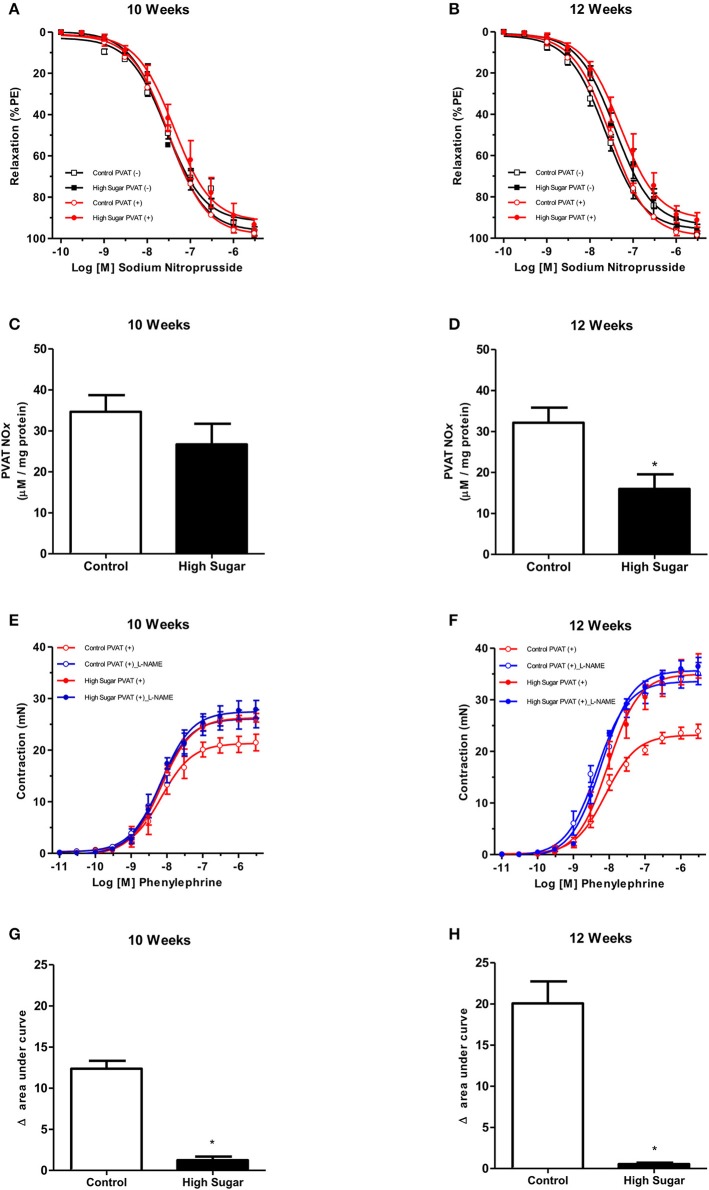
High sugar diet decreases nitric oxide (NO) generation in the PVAT. Concentration-response curves to sodium nitroprusside, a NO donor, were performed in endothelium-denuded aortic rings, without (–) or with (+) PVAT, from rats fed the control (*n* = 7) or high sugar (*n* = 7) diet for **(A)** 10 or **(B)** 12 weeks. Nitrite and nitrate (NOx) formation in the PVAT of rats fed the control (*n* = 6) or high sugar diet (*n* = 6) for **(C)** 10 or **(D)** 12 weeks. Concentration-response curves to phenylephrine were performed in endothelium-denuded aortic rings, without (–) or with (+) PVAT, from rats fed the control (*n* = 7) or high sugar (*n* = 7) diet for **(E)** 10 or **(F)** 12 weeks, incubated with the NO synthase inhibitor, L-NAME (10^−4^ M, for 30 min) or vehicle. **(G,H)** show the delta of the areas under the curves to phenylephrine in the presence of PVAT after incubation with L-NAME or vehicle. Data are represented as the mean ± SEM. Student's *t*-test: ^*^p < 0.05 High Sugar vs. Control.

**Figure 5:**
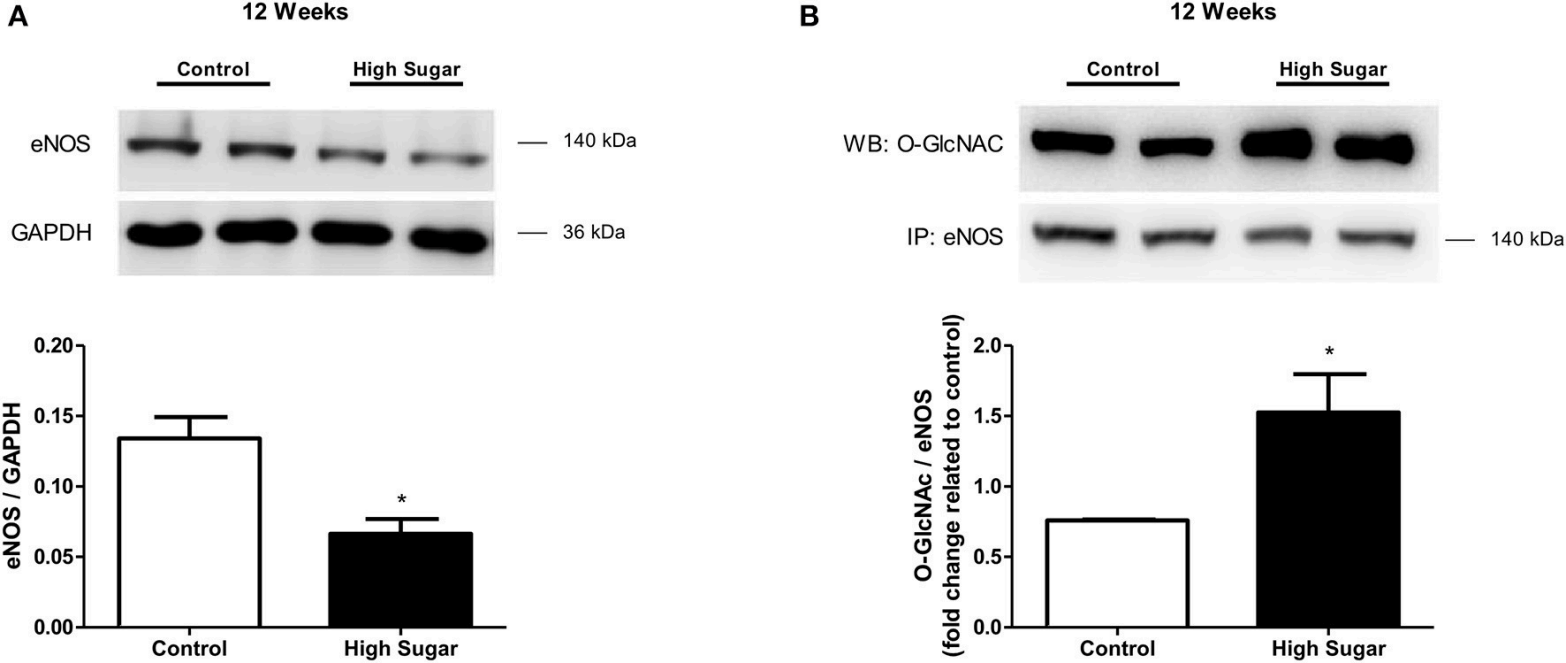
High sugar diet decreases endothelial nitric oxide synthase (eNOS) in the PVAT. **(A)** eNOS protein expression in aortic PVAT from rats fed the control (*n* = 4) or high sugar (*n* = 4) diet for 12 weeks, determined by western blot analysis, and **(B)** *O*-GlcNAc-modified eNOS, determined by immunoprecipitation of eNOS and probing with the anti-*O*-GlcNAc antibody. Data are represented as the mean ± SEM. Mann Whitney test: ^*^p < 0.05 High Sugar *vs*. Control.

Considering that NO is produced and released by the PVAT and induces relaxation of vascular smooth muscle cells (VSMC), and that PVAT dysfunction can be related to either changes in signaling pathways in the vasculature or to a defective production of NO by the PVAT, the effects of the high sugar diet on sodium nitroprusside (SNP)-induced endothelium-independent vascular relaxation as well as in NO bioavailability were determined. There was no difference in vascular relaxation to SNP in rats fed the high sugar diet either for 10 or 12 weeks (Figures 4A,B, Table 4), indicating that the high sugar diet does not reduce NO-mediated signaling in VSMC. NO formation in PVAT from rats fed the high sugar diet during 10 weeks was not decreased (Figure 4C). However, PVAT from high sugar diet-fed rats for 12 weeks exhibited decreased NO levels (Figure 4D). Interestingly, whereas L-NAME produced an upward shift in phenylephrine responses in endothelium-denuded and PVAT-intact aortas from rats fed the control diet, this effect was not observed in aortas from the high sugar-fed group, i.e., the NO synthase inhibitor L-NAME did not further enhance the effects of the high sugar diet [either for 10 weeks (Figure 4E, Table 4) or 12 weeks (Figure 4F, Table 4)] on the loss of PVAT anti-contractile effect. These data indicate that the high sugar diet decreases PVAT anti-contractile effect by reducing NO bioavailability (Figures 4G,H). Consistent with these results, the expression of endothelial nitric oxide synthase (eNOS) was significantly reduced in the PVAT from high sugar-fed rats (Figure 5A) and this change was accompanied by increased eNOS *O*-GlcNAcylation (Figure 5B). These results indicate that *O*-GlcNAcylation of eNOS impairs the activity of this enzyme and decreases NO production in PVAT.

**Table 4:** *p*D_2_ and Emax (mN) values of sodium nitroprusside-induced relaxation and phenylephrine-induced contraction in thoracic aorta arteries, from Control or High Sugar-fed rats, incubated with vehicle or L-NAME.

	***p*****D**_**2**_		**Emax**	
**Groups**	**10 Weeks**	**12 Weeks**	**10 Weeks**	**12 Weeks**
Control PVAT (–) *(Sodium nitroprusside)*	7.5 ± 0.05 (*n* = 6)	7.6 ± 0.05 (*n* = 6)	92.8 ± 2.0 (*n* = 6)	95.8 ± 1.9 (*n* = 6)
High Sugar PVAT (–) *(Sodium nitroprusside)*	7.6 ± 0.05 (*n* = 7)	7.4 ± 0.06 (*n* = 7)	96.4 ± 1.9 (*n* = 7)	94.7 ± 2.3 (*n* = 7)
Control PVAT (+) *(Sodium nitroprusside)*	7.4 ± 0.02 (*n* = 6)	7.5 ± 0.02 (*n* = 6)	97.1 ± 1.1 (*n* = 6)	98.1 ± 1.1 (*n* = 6)
High Sugar PVAT (+) *(Sodium nitroprusside)*	7.3 ± 0.08 (*n* = 7)	7.3 ± 0.07 (*n* = 7)	92.7 ± 2.0 (*n* = 7)	92.8 ± 2.9 (*n* = 7)
Control PVAT (+)	8.1 ± 0.09 (*n* = 6)	8.1 ± 0.05 (*n* = 6)	21.3 ± 0.7 (*n* = 6)	23.1 ± 0.4 (*n* = 6)
Control PVAT (+) *(L-NAME)*	8.1 ± 0.08 (*n* = 7)	8.3 ± 0.06 (*n* = 7)	26.1 ± 0.7 (*n* = 7)^+^	33.5 ± 0.7 (*n* = 7)^+^
High Sugar PVAT (+)	8.2 ± 0.04 (*n* = 6)	8.0 ± 0.07 (*n* = 6)	26.2 ± 0.4 (*n* = 6)	34.9 ± 0.9 (*n* = 6)
High Sugar PVAT (+) *(L-NAME)*	8.1 ± 0.06 (*n* = 7)	8.2 ± 0.04 (*n* = 7)	27.4 ± 0.6 (*n* = 7)	35.7 ± 0.5 (*n* = 7)

Data represent the mean ± SEM of n experiments. Two-way ANOVA:

+ *p < 0.05 vs. respective Control PVAT (+)*.

**Figure 6:**
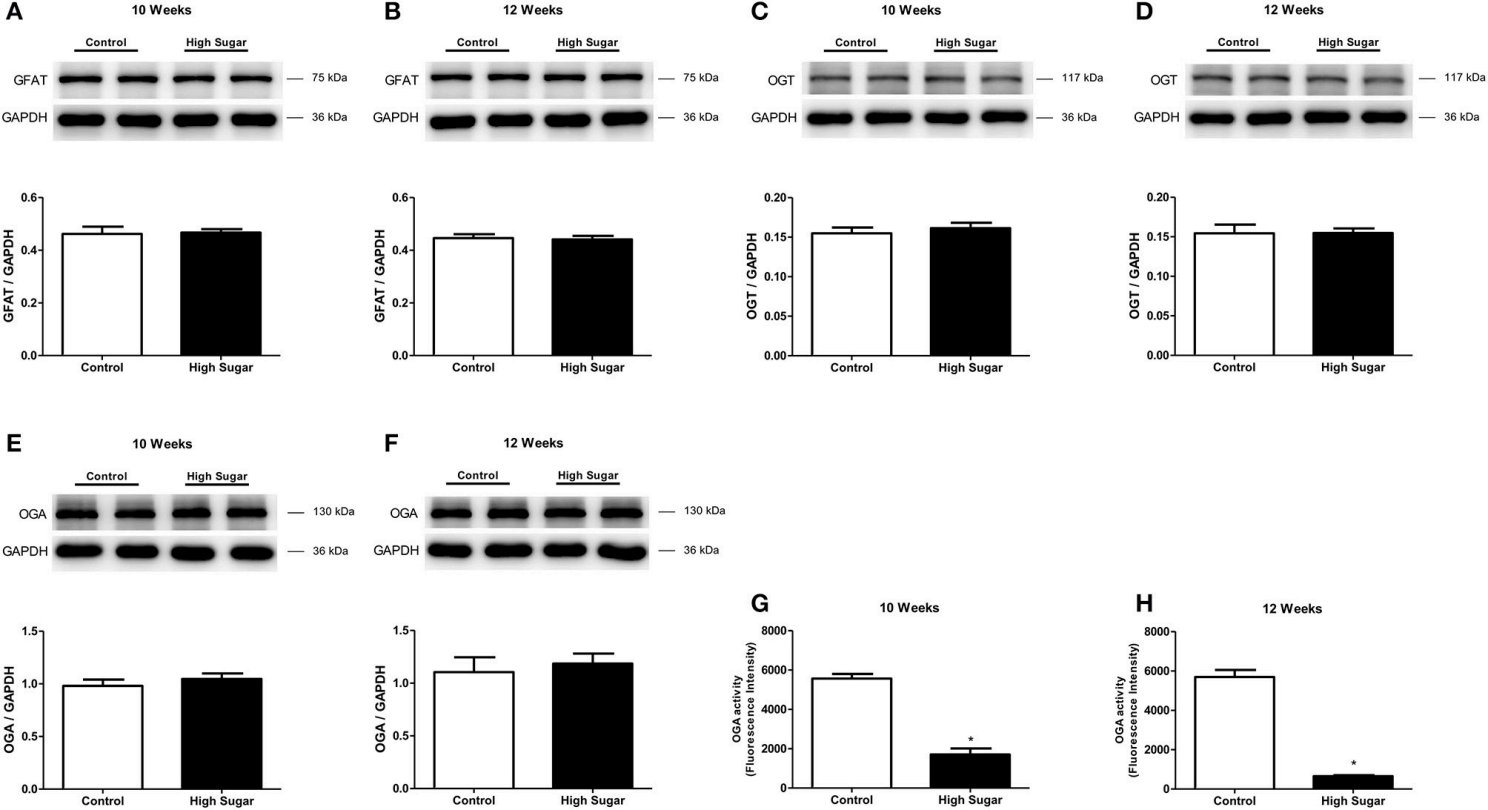
High sugar diet did not alter expression of proteins that regulate the hexosamine pathway, but decreases OGA protein activity. **(A,B)** GFAT, glucosamine-fructose-6-phosphate aminotransferase (*n* = 6); **(C,D)** OGT, *O*-linked *N*-acetylglucosamine (GlcNAc) transferase (*n* = 6); **(E,F)** OGA, *O*-GlcNAcase (*n* = 6) proteins expression, determined by western blot analysis. OGA activity in the PVAT from rats fed the control (*n* = 6) or high sugar (*n* = 6) diet for **(G)** 10 and **(H)** 12 weeks. Data are represented as the mean ± SEM. Student's *t*-test: ^*^p < 0.05 High Sugar vs. Control.

The effects of high sugar diet on the expression of proteins that directly regulate the *O*-GlcNAcylation process were also determined. No differences were detected in the PVAT expression of GFAT, the first and rate-limiting enzyme of the hexosamine biosynthesis pathway, between rats that received the control and high sugar diets for 10 (Figure 6A) or 12 (Figure 6B) weeks. Similarly, there were no differences in the expression of OGT (Figures 6C,D) or OGA (Figures 6E,F) enzymes in the PVAT from rats fed the control and high sugar diets for 10 or 12 weeks. However, OGA activity was significantly reduced in the PVAT of rats fed the high sugar diet for 10 (Figure 6G) and 12 (Figure 6H) weeks, suggesting that *O*-GlcNAc elevation in PVAT is a function of decreased OGA activity.

**Figure 7:**
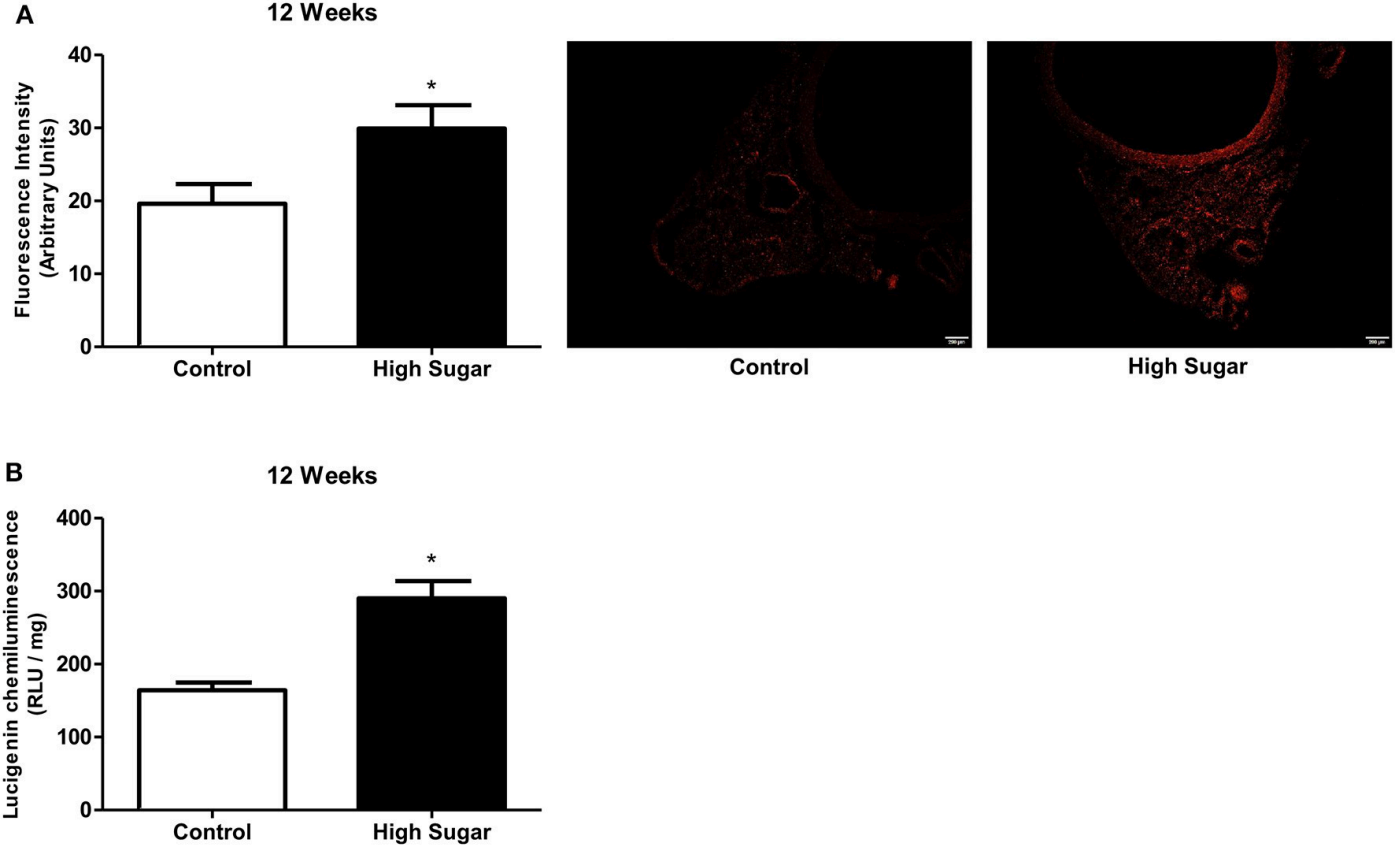
High sugar diet increases reactive oxygen species (ROS) generation in the PVAT. ROS generation, measured **(A)** by DHE (Dihydroethidium) and **(B)** by lucigenin in the PVAT from rats fed the control (*n* = 5) or high sugar (*n* = 5) diet for 12 weeks. Data are represented as the mean ± SEM. Student's *t*-test: ^*^p < 0.05 High Sugar vs. Control.

**Figure 8:**
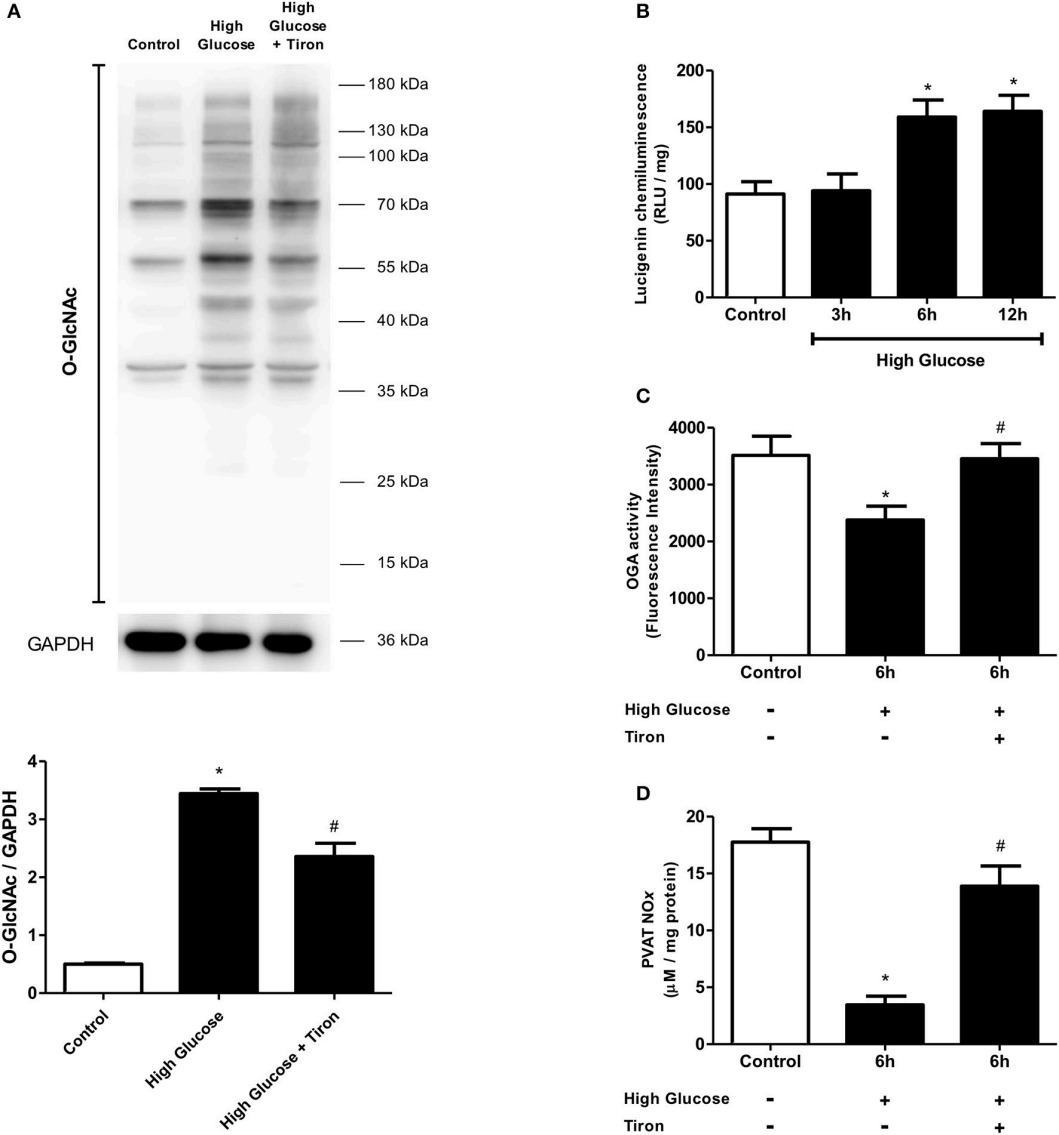
Effect of ROS in *O*-GlcNAcylation processes in PVAT incubated with high glucose medium. **(A)** *O*-GlcNAc-modified protein expression is increased in PVAT incubated with high glucose medium for 6 h and this increase is attenuated by simultaneous incubating with Tiron (10^−4^ M), a superoxide anion scavenger. **(B)** ROS production increases in the PVAT incubated in high glucose medium for 6 and 12 h. The PVAT maintained in high glucose medium for 6 h and incubated with Tiron does not exhibit **(C)** decreased OGA activity or **(D)** decreased NOx production. Data are represented as the mean ± SEM. Two-way ANOVA: ^*^p < 0.05 High Glucose vs. Control, ^#^*p* < 0.05 High Glucose + Tiron vs. High Glucose, *n* = 6 for each experimental group.

We have recently reported that increased mitochondrial ROS generation mediates the loss of PVAT anti-contractile effects in high-fat diet obese mice (da Costa et al., 2017). To explore the possibility that increased *O*-GlcNAc levels in the PVAT are linked to oxidative stress, we assessed the generation of NADPH-derived ${\text{O}}_{2}^{.-}$ by the lucigenin-enhanced chemiluminescence and DHE fluorescence techniques. ${\text{O}}_{2}^{.-}$ production was significantly increased in the PVAT of rats fed the high sugar diet for 12 weeks (Figures 7A,B). In view of the pro-oxidative phenotype observed in the PVAT, we hypothesized that the increased flux of glucose through the hexosamine biosynthetic pathway leads to increased *O*-GlcNAc modification of PVAT proteins via increased generation of NADPH-derived ${\text{O}}_{2}^{.-}$. To verify this hypothesis, PVAT from rats fed the control diet was exposed to high glucose medium for 6 h. Incubation in high glucose medium increased the PVAT content of *O*-GlcNAc-modified proteins, and effect that was attenuated by pre-incubation with Tiron (10^−4^ M) (Figure 8A). Similar to what was observed in the PVAT from rats fed the high sugar diet, the high glucose medium increased ROS generation (Figure 8B), which was accompanied by a decrease in OGA activity (Figure 8C). Reduction of OGA activity was also prevented by Tiron, suggesting that ROS modulate PVAT OGA activity. Finally, high glucose medium-induced modifications were followed by decreased PVAT NO production, which was prevented by the pre-incubation with Tiron (Figure 8D).

**Figure 9:**
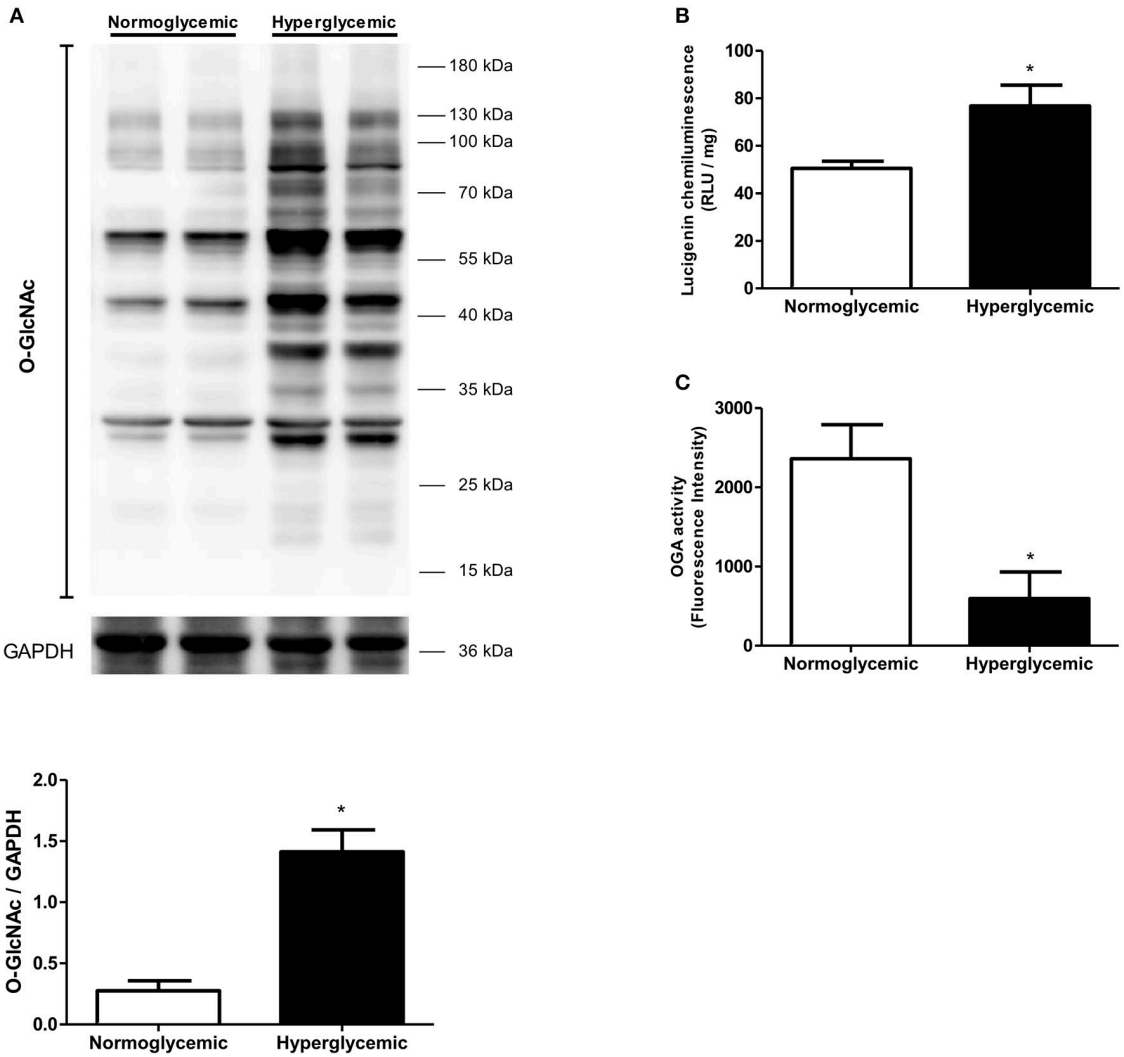
Effect of glycemia on *O*-GlcNAc-modified protein profile in human visceral adipose tissue. **(A)** *O*-GlcNAc-modified protein expression and **(B)** ROS generation are increased whereas **(C)** OGA activity is decreased in the adipose tissue of human with hyperglycemia. Data are represented as the mean ± SEM. Mann Whitney test: ^*^p < 0.05 Hyperglycemic vs. Normoglycemic, *n* = 4 for each group.

To determine whether there is a correlation between data from the experimental animal and the clinical onset, visceral adipose tissue samples from normoglycemic (blood glucose mean 92.5 mg/dL) and hyperglycemic (mean blood glucose 173.1 mg/dL) patients were analyzed. Increased levels of *O*-GlcNAc-modified proteins were found in the visceral adipose tissue from hyperglycemic individuals in comparison to samples from normoglycemic subjects. In addition, increased ROS generation (Figure 9B) and decreased OGA activity (Figure 9C) were detected in the visceral adipose tissue from hyperglycemic patients.

## Discussion

**Figure 10:**
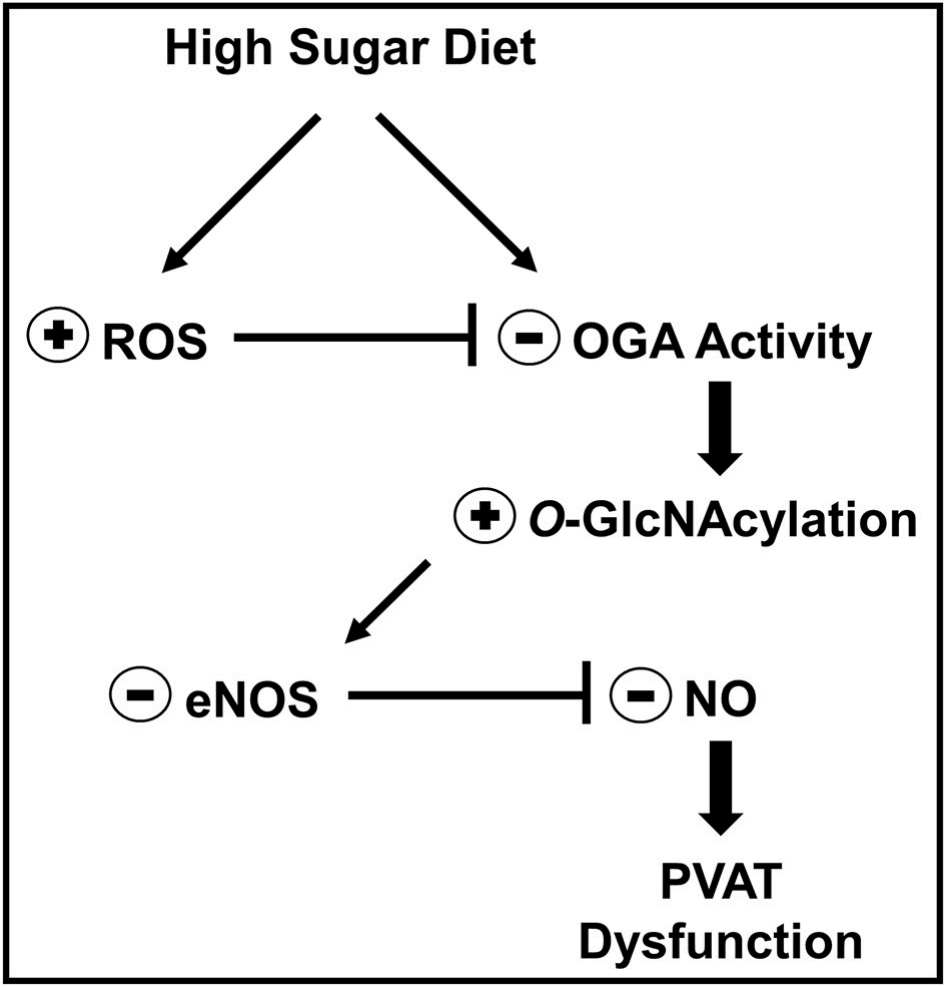
Mechanism proposed to explain how *O*-GlcNAc-modified proteins contribute to high sugar diet-induced PVAT dysfunction. In the context of hyperglycemia, such as in animals fed a high sugar diet or in hyperglycemic patients, increased PVAT ROS generation leads to inhibition of OGA activity and accumulation of *O*-GlcNAc-modified proteins, including eNOS. *O*-GlcNAcylation of eNOS reduces NO production by the enzyme and is linked to PVAT dysfunction.

PVAT is increasingly recognized as a widespread and relevant tissue in vascular biology as well as an important determinant of the cardiovascular complications associated with obesity and type 2 diabetes (Meijer et al., 2011). However, the molecular mechanisms underlying PVAT dysfunction in these conditions are largely unknown. Increased *O*-GlcNAcylation has been reported in human and animal visceral adipose tissue during pathological conditions such as hypertension, obesity, and type 2 diabetes (Ma and Hart, 2013). Nevertheless, the role of *O*-GlcNAcylation in PVAT dysfunction has not been previously determined. The present study demonstrates that *O*-GlcNAcylation plays a key role on high-sugar diet-induced PVAT dysfunction. Our studies reveal a previously unknown function of *O*-GlcNAcylation in controlling the anti-contractile activity of the PVAT. Chronic hyperglycemia increased ROS production and induced a phenotype of increased *O*-GlcNAcylation and decreased ADRF release, primarily by regulating NO production in the adipocytes (Figure 10). These new findings have important implications for prevention and treatment of vascular complications during obesity and other metabolic disorders and might, therefore, underscore O-GlcNAcylation as a target to combat vascular damage.

The results delineate potential negative consequences of increased PVAT *O*-GlcNAcylation in response to chronic hyperglycemia (Figure 2). Previous studies have demonstrated that acute increases in O-GlcNAcylation in 3T3-L1 adipocytes treated with PUGNAc lead to insulin resistance (Vosseller et al., 2002). Moreover, a similar phenomenon has been observed in rat skeletal muscle (Arias et al., 2004). However, few studies have examined the function of chronic increases in *O*-GlcNAcylation. Our findings, along with other studies (Medford et al., 2012; Heath et al., 2014), indicate that increased *O*-GlcNAcylation over an extended period, as observed in the later stages of diabetes, may cause adverse complications in the cardiovascular system (Figure 3). By using Thiamet-G, a potent and selective OGA inhibitor, our data provide further evidence that increased PVAT *O*-GlcNAcylation impairs the anti-contractile effects to phenylephrine, mediated by this tissue (Figure 1). In this case, the use of culture medium to keep the vessels for 24 h (time required to increase *O*-GlcNAc-modified proteins in PVAT) may be considered a methodological limitation. This *ex vivo* period changes the contractile properties when compared to freshly isolated vessels. However, there is no loss of information, since control vessels were also exposed to the same cultivation period.

Mechanistic studies demonstrated that increased PVAT *O*-GlcNAcylation following chronic hyperglycemia is linked to inhibition of OGA activity. Consistent with clinical observations demonstrating an association between PVAT dysfunction and other vascular complications in obesity and diabetes, our findings demonstrate increased aortic contraction in high sugar-fed rats, which was further worsened in the presence of PVAT (Figure 3). Our data also demonstrate increased *O*-GlcNAcylation associated with OGA inhibition in human visceral adipose tissue following long-term hyperglycemic conditions (Figure 9). Together, these studies indicate a causative link between increased *O*-GlcNAcylation in the PVAT and hyperglycemia-induced vascular dysfunction.

PVAT from high sugar-fed rats displays a significant reduction in NO production (Figure 5) associated with decreased eNOS expression (Figure 4). Previous studies have shown eNOS expression in adipose tissues (Ribiere et al., 1996; Victorio et al., 2016) and adipocytes (Ribiere et al., 1996). It is assumed that adipocyte-derived NO is released into the interstitial fluid and diffuses into the capillaries and adjacent arterioles causing vasodilation (Mastronardi et al., 2002). Indeed, enhanced relaxant response of mesenteric arteries and increased leptin-mediated NO production in the mesenteric artery PVAT have been demonstrated at the early phase of diet-induced obesity in C57BL/6J mice (Gil-Ortega et al., 2010). In addition, the studies of Xia et al. confirmed that PVAT-derived NO contributes to acetylcholine-induced vasodilation (Xia et al., 2016). The authors demonstrated that, under diet-induced obesity conditions, eNOS-driven NO production is reduced in the PVAT. In our model, the loss of the PVAT anti-contractile function in rats fed a high sugar diet was not further modified by eNOS inhibition. Thus, eNOS-derived NO in PVAT plays a major role in the anti-contractile effects mediated by PVAT. The intrinsic relaxant responsiveness of VSMC was preserved, since sodium nitroprusside-induced relaxations were identical despite the loss of eNOS-derived NO in PVAT (Figure 4). These data suggest that changes in PVAT anti-contractile effects during hyperglycemia are most likely caused by alterations in PVAT-derived components and not by changes in the VSMC function.

Notably, the present study provides novel mechanisms underlying the regulation of eNOS activity in the PVAT. Although impaired activation of PVAT eNOS and reduction of PVAT-derived NO have been associated with obesity-induced vascular dysfunction, whether eNOS activation in PVAT is regulated directly by *O*-GlcNAcylation is not clear. Our studies have identified that decreased eNOS expression is accompanied by an increase in eNOS *O*-GlcNAcylation, which promotes vascular dysfunction during hyperglycemia (Figure 5). Increased *O*-GlcNAcylation of eNOS, which is associated with decreased phosphorylation at Ser^1177^, the site responsible for activation of the enzyme, has been reported in bovine aortic endothelial cells (Du et al., 2001). The work by Federici et al. (2002) also demonstrated an inverse correlation between eNOS *O*-GlcNAcylation and phosphorylation status in human coronary artery endothelial cells. Furthermore, the authors showed that both hyperglycemia and direct activation of the hexosamine biosynthesis pathway by glucosamine determines reduction in insulin-stimulated phosphorylation of eNOS by increased *O*-GlcNAcylation of key signaling molecules. Interestingly, Musicki et al. (2005) demonstrated that increased *O*-GlcNAcylation of eNOS-Ser^1177^ attenuates shear stress-induced increase in penile blood flow during diabetes. More recently, Beleznai and Bagi (2012) reported that increased *O*-GlcNAcylation contributes to impaired NO-mediated arteriolar dilation following hyperglycemia. Overall, our findings show that increased *O*-GlcNAcylation of eNOS impairs the activity of this enzyme and the subsequent NO production in the PVAT and support the concept that PVAT *O*-GlcNAcylation may be a significant contributing factor to vascular dysfunction in metabolic syndrome.

Apart from inhibiting eNOS, basal *O*-GlcNAcylation may regulate other signaling systems that decrease NO production, such as ROS (Lima et al., 2012). In the present study, acute and chronic high glucose increased PVAT generation of ROS. *O*-GlcNAc may influence the expression or function of a variety of proteins involved in high glucose-induced ROS production. However, it is equally plausible that much of the effect of high glucose levels on PVAT *O*-GlcNAcylation is secondary to the increase in PVAT ROS generation. In addition to modulating the activity of specific intracellular protein kinases and phosphatases (Takakura et al., 1999; Weber et al., 2004), ROS stimulate the hexosamine biosynthetic pathway and, consequently, *O*-GlcNAcylation (Brownlee, 2001). For instance, increased production of mitochondria-derived ROS induces both changes in phosphorylation and *O*-GlcNAcylation of many intracellular proteins (Jones et al., 2008; Laczy et al., 2009). Restoration of both OGA activity and NO production by Tiron (Figure 8) further supports a role for ROS-mediated signals in mediating *O*-GlcNAcylation-induced impairment of eNOS activity and vascular dysfunction. The concurrent increase in both ROS generation and *O*-GlcNAcylation clearly demonstrate the reciprocal regulation between *O*-GlcNAcylation and ROS. Nevertheless, our findings, along with the western blot data showing that Tiron only partially reduces PVAT *O*-GlcNAc levels (Figure 8), suggest that chronic hyperglycemia increases *O*-GlcNAcylation of target proteins in the PVAT via increased ROS generation.

As discussed above, the different outcomes of reducing ROS formation probably reflect the involvement of different signal transduction pathways in high glucose-treated PVAT. Reinforcing this view, it has been reported that both oxidative stress and high glucose decrease endothelial NO bioavailability, impair vascular relaxation (King, 1996; Creager et al., 2003; Landmesser et al., 2003), and increase *O*-GlcNAcylation (Lima et al., 2012; Ma and Hart, 2013). Further studies are warranted to dissect the precise signaling cascades that are responsible for hyperglycemia-induced *O*-GlcNAcylation of PVAT eNOS. However, the novel regulation of eNOS activation in PVAT by *O*-GlcNAcylation uncovered in this study may not only impact the biological function of this tissue, but also provides novel mechanistic insights into the pathogenesis of hyperglycemia-associated vascular disease.

In summary, the present study demonstrates a novel causative link between chronic increases in *O*-GlcNAcylation of target proteins in the PVAT and hyperglycemia-associated vascular dysfunction. It also provides a novel molecular insight into the mechanism underlying the regulation of eNOS activity, with *O*-GlcNAcylation leading to reduced NO production and PVAT anti-contractile effect. Our studies also reveal a critical interplay between *O*-GlcNAcylation and ROS generation in the PVAT, which mediates high glucose-induced PVAT dysfunction and vascular injury. These findings have determined *O*-GlcNAcylation as a novel contributor to the process of hyperglycemia-induced PVAT dysfunction and identified *O*-GlcNAcylation of eNOS as a possible target for the development of therapies for vascular dysfunction in metabolic syndrome.

## Author contributions

RdC, JdS, NdSL, and RT participated in the design of the study; RdC, JdS, JA, TD, DR, and LG conducted the experiments; RT and NdSL contributed new reagents or analytical tools; RdC, JdS, JA, NdSL, and RT performed the data analysis; RdC, JdS, NdSL, and RT wrote the paper.

### Conflict of interest statement

The authors declare that the research was conducted in the absence of any commercial or financial relationships that could be construed as a potential conflict of interest.
